# Medical Attendance in Cottage Hospitals

**Published:** 1912-03-23

**Authors:** Jacob Wakefield

**Affiliations:** Sedgwick House, Kendal.


*To the Editor of* THE HOSPITAL.

### MEDICAL ATTENDANCE IN COTTAGE HOSPITALS.

*To the Editor of* THE HOSPITAL.

SIR,—In THE HOSPITAL of March 16 I notice a paragraph under "Institutional News," headed "Wood Green Hospital Dispute."

At the end it is stated "The right remedy is to allow all doctors to attend their own patients here as *elsewhere.*" May I ask what is meant in this case as "elsewhere"? I presume the writer doesn't mean to suggest that this is a custom prevailing at many hospitals? Possibly the statement is meant to refer to Cottage Hospitals only; but even so, it would seem somewhat difficult for eighteen doctors to have the run of a hospital, unless, of course, it was such a small one that the cases were practically in the care of only three or four of this large body of medical men.

I am a regular reader of your paper, which must be my excuse for venturing to ask you about this.—Yours very truly, JACOB WAKEFIELD.

Sedgwick House, Kendal.

[Elsewhere means in cottage hospitals. The eighteen would only visit any of their own patients who happened to be in hospital. Thus only three or four doctors would be likely to attend on any one day.—ED., THE HOSPITAL.]

